# Potential of selected Senegalese *Aedes spp*. mosquitoes (*Diptera*: *Culicidae*) to transmit Zika virus

**DOI:** 10.1186/s12879-015-1231-2

**Published:** 2015-11-02

**Authors:** Cheikh Tidiane Diagne, Diawo Diallo, Oumar Faye, Yamar Ba, Ousmane Faye, Alioune Gaye, Ibrahima Dia, Ousmane Faye, Scott C. Weaver, Amadou Alpha Sall, Mawlouth Diallo

**Affiliations:** Unité d’Entomologie Médicale, Institut Pasteur de Dakar, 36 Avenue Pasteur, BP 220, Dakar, Senegal; Unité des Arbovirus et Virus de Fièvres Hémorragiques, Institut Pasteur de Dakar, 36 Avenue Pasteur, BP 220, Dakar, Senegal; Département de Biologie Animale, Laboratoire d’Écologie Vectorielle et Parasitaire, Université Cheikh Anta Diop, Dakar, Senegal; Institute for Human Infections and Immunity, Center for Biodefense and Emerging Infectious Diseases, University of Texas Medical Branch, Galveston, TX 77555–0610 USA; Department of Pathology and Microbiology & Immunology, University of Texas Medical Branch, Galveston, TX 77555–0610 USA

**Keywords:** *Aedes*, Arboviruses, Oral infection, Senegal, Vector competence, West Africa, Zika virus

## Abstract

**Background:**

Zika virus (ZIKV; genus *Flavivirus*, family *Flaviviridae*) is an emerging virus of medical importance maintained in a zoonotic cycle between arboreal *Aedes spp*. mosquitoes and nonhuman primates in African and Asian forests. Serological evidence and virus isolations have demonstrated widespread distribution of the virus in Senegal. Several mosquito species have been found naturally infected by ZIKV but little is known about their vector competence.

**Methods:**

We assessed the vector competence of *Ae. aegypti* from Kedougou and Dakar*, Ae. unilineatus, Ae. vittatus* and *Ae. luteocephalus* from Kedougou in Senegal for 6 ZIKV strains using experimental oral infection. Fully engorged female mosquitoes were maintained in an environmental chamber set at 27 ± 1 °C and 80 ± 5 % Relative humidity. At day 5, 10 and 15 days post infection (dpi), individual mosquito saliva, legs/wings and bodies were tested for the presence of ZIKV genome using real time RT-PCR to estimate the infection, dissemination, and transmission rates.

**Results:**

All the species tested were infected by all viral strains but only *Ae. vittatus* and *Ae. luteocephalus* were potentially capable of transmitting ZIKV after 15 dpi with 20 and 50 % of mosquitoes, respectively, delivering epidemic (HD 78788) and prototype (MR 766) ZIKV strains in saliva.

**Conclusion:**

All the species tested here were susceptible to oral infection of ZIKV but only a low proportion of *Ae. vittatus* and *Ae. luteocephalus* had the viral genome in their saliva and thus the potential to transmit the virus. Further investigations are needed on the vector competence of other species associated with ZIKV for better understanding of the ecology and epidemiology of this virus in Senegal.

**Electronic supplementary material:**

The online version of this article (doi:10.1186/s12879-015-1231-2) contains supplementary material, which is available to authorized users.

## Background

Zika virus (ZIKV; genus *Flavivirus*, family *Flaviviridae*) is an emerging globally mosquito-borne pathogen of growing public health importance. The virus was first isolated in 1947 from a febrile sentinel rhesus monkey and one year later from *Ae. africanus* in Uganda [1]. Non-human primates (NHPs) were implicated as the vertebrates hosts of ZIKV in Africa and Asia [2]. The first well-documented report of human ZIKV infection was in Uganda in 1964 when Simpson described his own occupationally acquired illness [3]. Subsequently, ZIKV has been recognized to be a cause of febrile illness in humans in Africa and Southeast Asia with symptoms including fever, headache, conjunctivitis, myalgia, rash, joint pains [4–6]. Serological evidence and virus isolations have demonstrated widespread distribution of the virus in Africa, the Indian subcontinent, Southeast Asia and most recently Micronesia and French Polynesia [3, 7–11]. The implication of *Ae. aegypti* in the urban transmission of ZIKV came first from field evidence including the high prevalence of anti-ZIKV antibodies in the urban population of Nigeria [12], the coincidance of peaks of human ZIKV infections and *Ae. aegypti* population in Indonesia [7] and the isolation of the virus from a pool of *Ae. aegypti* in Malaysia [13]. This implication of *Ae. aegypti* was confirmed by early experimental studies wich demonstrated the competence of this species to transmit ZIKV [14, 15]. The isolation of the virus from a pool of *Ae. aegypti* in Malaysia provided the first evidence of ZIKV transmission outside Africa.

ZIKV was also isolated from *Ae. africanus* and *Ae. apicoargenteus* in Uganda and the Central African Republic [16, 17]; from *Ae. luteocephalus* in Nigeria in 1969 and 1972 [12]; and from *Ae. vittatus*, *Ae. furcifer*, and *Ae. aegypti* in Cote d’Ivoire in 1999 [18].

In Senegal, the first evidence of ZIKV circulation was the isolation of a strain from *Ae. luteocephalus* collected in 1968 in the Saboya forest, 187 km from Dakar, in the western part of the country [15]. One year later, the virus was isolated from *Ae. luteocephalus*, *Ae. furcifer*-*taylori* and *An. gambiae s.l*., and a human in Bandia located 65 km from Dakar. In Kedougou, Southeastern Senegal, 381 ZIKV isolates were collected as part of an entomological surveillance programme from 1972 to 2011, mainly from *Ae. africanus, Ae. luteocephalus, Ae. furcifer,* and *Ae. taylori*, 7 times from humans and twice from NHPs (*Cercopithecus aethiops*, *Erythrocebus patas*). Serological studies in 1988 and 1990 in the area showed that 10.1 and 2.8 % of humans had Immunoglobuline M (IgM) antibodies to ZIKV [9].

Although several mosquito species have been found naturally infected by ZIKV, little is known about their vector competence. Hence, the purpose of the present study was to investigate vector competence of populations of *Ae. aegypti* from Dakar and Kedougou and *Ae. unilineatus, Ae. vittatus*, and *Ae. luteocephalus* from Kedougou for 6 ZIKV strains to take into account the high diversity of hosts (human, NHPs, and several mosquito species) and location from which ZIKV strains were isolated.

## Methods

### Ethical approval

The protocol of this study has been approved by the Senegalese National Ethic Committee under protocol SEN29/08; 2472/MSP/DS/DER. Because this study was done in collaboration with a team from the University of Texas Medical Branch (UTMB), the UTMB Institutional Animal Care and Use Committee also approved the animal experiments under protocol 02-09-068. UTMB complies with all applicable regulatory provisions of the U.S. Department of Agriculture (USDA)-Animal Welfare Act; the National Institutes of Health (NIH), Office of Laboratory Animal Welfare-Public Health Service (PHS) Policy on Human Care and Use of Laboratory Animals; the U.S. Government Principles for the Utilization and Care of Vertebrate Animals Used in Research, Teaching, and Testing developed by the Interagency Research Animal Committee (IRAC), and other federal statutes and state regulations relating to animal research. The animal care and use program at UTMB conducts reviews involving animals in accordance with the *Guide for the Care and Use of Laboratory Animals* (2011) published by the National Research Council.

### Mosquitoes

Table 1 describes the characteristics and geographic origin of the populations of *Ae. aegypti*, *Ae. unilineatus*, *Ae. vittatus*, and *Ae. luteocephalus* tested in this study. These species were chosen taken into account their abundance, anthrophophilic behaviour and association to ZIKV in the field [19]. For each population, several breeding habitats were prospected and collected eggs reared in the laboratory. Females F0 were fed several times on guinea pigs to obtain F1 generation eggs. These eggs were hatched and the larvae reared to F1 adults used in this study. This F1 generation were maintained exclusively with a 10 % sucrose solution at 27 °C, 80 % relative humidity (RH), 16:8 h (L:D) photoperiod.

**Table 1:** Mosquito species tested for vector competence for Zika virus

Species	Source^a^	Habitat	Generation
*Ae. aegypti*	Dakar	Domestic	F_1_
	Kedougou	Sylvatic	F_1_
*Ae. unilineatus*	Kedougou	Sylvatic	F_1_
*Ae. vittatus*	Kedougou	Sylvatic	F_1_
*Ae. luteocephalus*	Kedougou	Sylvatic	F_1_

^a^All mosquitoes were collected during 2012

### Virus strains

Hosts origin, year of collection and passage histories of the six ZIKV strains used in this study are presented in Table 2. To prepare viral stocks, ZIKV isolates were amplified in *Ae. albopictus* C6/36 cells for a week at 27 °C and infection progression was monitored using indirect immunofluorescence assay. Supernatant fluids were collected and viral titers estimated by serial 10-fold dilutions on Vero cells [Plaque forming unit (PFU)/ml] as previously described by De Madrid and Porterfield [20]. Each virus stock was divided into 500 μl aliquots and stored at - 80 °C until use.

**Table 2:** Zika virus strains used for this study

ZIKV strains	Host origin	Year of collection	Location	Passage history^a^
ArD 128000	Mosquito (*Ae. luteocephalus*)	Oct. 11^th^ 1997	Kedougou (Senegal)	6
ArD 132912	Mosquito (*Ae. dalzieli*)	Nov. 20^th^ 1998	Kedougou (Senegal)	4
ArD 157995	Mosquito (*Ae. dalzieli*)	Nov. 17^th^ 2001	Kedougou (Senegal)	6
ArD 165522	Mosquito (*Ae. vittatus*)	Oct. 21^st^ 2002	Kedougou (Senegal)	5
HD 78788	Human blood	Feb. 14^th^ 1991	Dakar (Senegal)	Unknown
Ref. (MR 766)	Monkey	Apr. 18^th^ 1947	Zika Forest (Uganda)	20

^a^Passages were conducted with *Aedes* (*Stegomyia*) *pseudoscutellaris* 61 cells (AP-61)

### Oral infection of mosquitoes

Mosquito infection has been performed according to procedures already described [21]. Briefly, one-week-old females of each mosquito species that have never taken blood meal were starved for 24 h before the infectious meal. These females were allowed to feed through a chicken skin membrane by the artificial feeding method described by Rutledge et al. [22]. The infectious meal consisted of two-thirds washed rabbit erythrocytes mixed with sucrose and fetal bovine serum (FBS) and one-third viral suspension. Adenosine 5’-triphosphate (ATP) was added at a final concentration of 5 × 10^−3^ M as phagostimulant. For each infection experiment, a sample of the virus-blood suspension was taken at the end of the mosquitoes feeding and stored at - 80 °C for titration as previously described [20]. After 30 min of exposure, Mosquitoes were cold anaesthetized and sorted according to their stomach repletion. Fully engorged specimens were preserved and maintained at 27 °C, 80 % RH, 16:8 h (L:D) photoperiod and fed on 10 % sucrose for extrinsic incubation of the virus.

At 5, 10, and 15 dpi, samples of mosquitoes were collected randomly, cold anesthesized, and their legs and wings removed and transferred individually into separate tubes. The proboscis of each mosquito was then inserted into a capillary tube containing 1 μL of FBS for salivation for up to 30 min. After salivation, each mosquito body (whole body except legs and wings) and saliva sample was placed in a separate tube. *Aedes luteocephalus* was tested only at 15 dpi because this species is difficult to rear, feed and maintain in the laboratory, so a small sample was available.

### Virus detection

Mosquito bodies, and legs/wings were triturated using cold pestles in 500 μl of L-15 medium (GibcoBRL, Grand Island, NY, USA). After trituration, pools were centrifuged at 7500 rpm for 10 min at 4 °C. For each sample, 100 μl of supernatant were used for RNA extraction with the QiaAmp Viral RNA Extraction Kit (Qiagen, Heiden, Germany) according to the manufacturer’s protocol with slight modification [23]. The RNA was amplified using a real-time RT-PCR assay and an ABI Prism 7500 SDS Real-Time apparatus (Applied Biosystems, Foster City, CA) using the QuantiTect kit (Qiagen, Hilden, Germany). The 25 μl reaction volume contained 5 μl of extracted RNA, 10 μl of buffer (2x QuantiTect Probe), 6.8 μl of RNase free water, 1.25 μl of each primer, 0.5 μl of probe, and 0.2 μl of enzymes. The primers and probe sequences were described by Faye et al. [23].

Only RT-PCR was used to detect ZIKV because the objective of this study is only to show the competence of the vector. Thus, if we are able to show that the virus reached the saliva, it implies that the vector is competent. In our previous experiences with other viruses (West Nile, Usutu), we have noticed that RT-PCR and infectious viral particles are generally very consistent and concordant in their conclusions and trends [24, 25]. Such a trend has also been confirmed on C6/36 cells for Chikungunya virus by Chen et al. [26].

### Data analysis

Infection (number of positive bodies/total number of mosquitoes tested), dissemination (number of infected legs & wings/total number of infected bodies), and transmission (number of positive saliva/number of infected legs & wings) rates were calculated for each species on each dpi. Rates were compared using Fisher’s exact test. The associations between viral titers and infection rates were assessed by Spearman’s rank-order correlation test. Differences were considered statistically significant at *p* < 0.05. Statistical tests were performed using R v. 3.0.1 (R Foundation for Statistical Computing, Vienna, Austria).

## Results

**Fig. 1:**
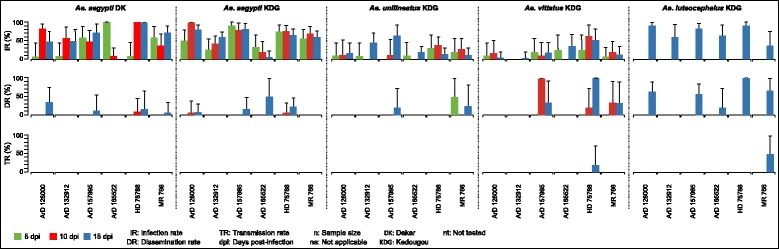
Infection, dissemination and transmission rates at different incubation period for different mosquito species orally exposed with six zika virus strains

After exposure to virus titers ranging from 2.7 × 10^6^ to 4 × 10^7^ PFU/ml (Additional file 1: Table S1), overall, 111 (50.2 %) of the 221 *Ae. aegypti* from Dakar, 216 (57.6 %) of the 375 *Ae. aegypti* from Kedougou, 56 (18.7 %) of the 300 *Ae. unilineatus*, 37 (14.4 %) of the 256 *Ae. vittatus* and 45 (75.0 %) of the 60 *Ae. luteocephalus* tested were infected by the six ZIKV (Fig. 1). No correlation was found between viral titer and mosquito infection rates at 5, 10 and 15 dpi (spearman test; rho > - 0.18, *p* > 0.95). Infection rates varied significantly between strain for each species tested at 5, 10 and 15 dpi (*p* < 0.05). Highest infection rates were generally observed at 10 dpi for *Ae. aegypti* from Dakar (3/6 viral strains), *Ae. aegypti* from Kedougou (4/6 viral strains) and *Ae. vittatus* (3/6 viral strains) and 15 dpi for *Ae. unilineatus* (4/6 viral strains). *Aedes aegypti*, *Ae. unilineatus*, and *Ae. vittatus* populations showed a decreasing trend in infection rates either from 5 to 10 dpi or from 10 to 15 dpi in 19 of the 24 infection assays performed.

The overall trend has been the low dissemination in the populations of *Ae. aegypti* from Dakar (6.3 % of the 111 specimen tested), *Ae. aegypti* from Kedougou (5.6 % of the 216 specimen tested) and *Ae. unilineatus* (5.3 % of the 56 specimen tested) and a relatively high dissemination rates in *Ae. vittatus* (27.0 % of the 37 specimen tested) and *Ae. luteocephalus* (42.2 % of the 45 specimen tested). Only *Ae. unilineatus* disseminated one viral strain at 5 dpi. Dissemination rates varied between 0 and 10 % for the populations of *Ae. aegypti* and beteween 0 and 100 % for *Ae. vittatus* at 10 dpi. At 15 dpi, these dissemination rates varied between 0 and 50 % for the populations of *Ae. aegypti* and beteween 0 and 100 % for *Ae. vittatus* and *Ae. luteocephalus.* Dissemination rates were statistically comparable for all species at 10 and 15 dpi (*p* > 0.09) except *Ae. luteocephalus* at 15 dpi (*p* = 0.03). The 2 populations of *Ae. aegypti* and *Ae. luteocephalus* disseminated 4 of the 6 ZIKV strains tested, *Ae. vittatus* 3 strains and *Ae. unilineatus* only 2 strains.

Taken into account all the six viral strains, a total of 7, 12, 3, 10 and 27 saliva of *Ae. aegypti* from Dakar, *Ae. aegypti* from Kedougou, *Ae. unilineatus*, *Ae. vittatus* and *Ae. luteocephalus,* respectivly were tested for the presence of ZIKV RNA. Only *Ae. vittatus* and *Ae. luteocephalus* transmitted ZIKV strains HD 78788 (20 %) and MR 766 (50 %) respectively at 15 dpi indicated by the presence of the viral genome in the mosquito saliva. These transmission rates were comparable (*p* = 1).

## Discussion

Only RT-PCR was used to detect ZIKV because the objective of this study is only to show the competence of the vector. Thus, if we are able to show that the virus reached the saliva, it implies that the vector is competent. Mean Ct values decreased gradually from 5 to 15 dpi for most infected species and ZIKV strain association suggesting that the mean amount of virus in each mosquito has increased and thus the RT-PCR was able to detect the ZIKV amplification (Additional file 1: Table S1).

All mosquito species tested were susceptible to ZIKV infection with infection rates varying according to viral strains and extrinsic incubation periods. In some cases, these rates were as high as those obtained with *Ae. aegypti* and *Ae. albopictus* populations from Singapore [27].

Our results also revealed globally in *Ae. aegypti*, *Ae. unilineatus*, and *Ae. vittatus* populations a decreasing trend in infection rates either from 5 to 10 dpi or from 10 to 15 dpi. Indeed, in 24 infection assays performed, 17 showed this profile. Similar profile were also observed in *Ae. aegypti* populations from Singapore between 6 and 7 dpi and those of *Ae. albopictus* from 6 to 10 dpi [27, 28]. This decrease following high infection rates obtained at 5 or 10 dpi suggest that ZIKV infection could induce an immune response that can lead to replication of the virus at undetectable levels.

The main findings of our study was the low dissemination and lack of transmission of the population of *Ae. aegypti* tested here. This result was unexpected taken into account very high transmission rates obtained in previous studies with this species in Senegal and Asia. Indeed, a previous study had shown that *Ae. aegypti* from Kebemer (a locality near Dakar, Senegal), were competent to ZIKV with a transmission rate of 88 % at 7 dpi [15], reaching as high as 95 % at 21 dpi. However, intrathoracic inoculation and transmission to newborn mice used in that study were different from our methods. With other viruses, intrathoracic inoculation is well known to bypass the midgut infection barrier, leading to a shorter period of extrinsic incubation, a direct exposure of the virus to the salivary glands, and thereafter generally more efficient infection and transmission [29].

The recent studies conducted on *Ae. aegypti* populations from Singapore using an oral infection methods similar to our method have generated high salivary glands infection rates of 62 and 100 % at 5 and 10 dpi, respectivly [27].

The low or lack of transmission we observed could not be attributed to the transmission method used (salivation on capillary tubes) which efficacy has been proven. Indeed, this system has been recently used to demonstrate vector competence of *Ae. albopictus* for the same ZIKV [28]; but also with various other mosquito species tested for various viruses [21, 30]. In addition, a comparative study has shown that *Ae. albopictus* and *Ae. taeniorhynchus* transmit better with the method of capillary tubes than with the use of an animal for transmission monitoring [31]. Because the experiments were carried out at 27 °C, the average temperature in Senegal during ZIKV transmission, the temperature may not be impacting our transmission results. Also the titers of the ZIKV we used could not be responsible because a virus suspension at a final concentration of 7.0 log_10_ tissue culture infectious dose (TCID_50_)/ml were sucessfully used in previous mosquito experiments [27, 28]. Our low transmission results were also not due to the detection method, since the real-time RT-PCR has often greater sensitivity than the other methods [32].

The difference between the transmission rates observed in this study and the others could be explained by genetic variability between *Ae. aegypti* populations from different geographical origin. The impact of this variability in vector competence results was demonstrated for several vector/virus associations [33, 34]. Although the lack of transmission by *Ae. aegypti* populations was not expected, this is consistant with the low number of ZIKV strains isolated from this species in West Africa. Indeed only 2 strains of ZIKV were isolated from this species in Senegal, one strain in Burkina Faso and one in Ivory Coast [35, 36].

The low competence of *Ae. vittatus* and *Ae. luteocephalus* is discordant with the high abundance of these species and their frequent association with ZIKV in the field [19, 35, 36]. Indeed, *Ae. vittatus*, with 22.98 % of the fauna, was the most abundant mosquito species collected by human landing catch in the Kedougou area between June 2009 and December 2010. This species has beeen found associated with ZIKV in Ivory Cost (2 strains) and Senegal (15 isolates at least) in West Africa. *Ae. luteocephalus* is generally less abundant than *Ae. vittatus* but more ZIKV was isolated in this species in Burkina Faso (40 strains), Ivory Cost (48 strains) and Senegal (92 strains).

However, an *Ae. aegypti* population with poor vector competence but high density have been shown to be the principal vector of a Yellow Fever outbreak in Nigeria [37].

The low infection and dissemination rates and the lack of transmission by *Ae. unilineatus* are in agreement with the single isolation of ZIKV from this species in nature and its low representation from human landing catch collection [38].

Our results are discordant with the pattern of ZIKV transmission in Southeastern Senegal. Indeed ZIKV has the highest frequency of detections among arboviruses found in this area. Its amplifications have been detected during 20 of the 34 years of monitoring that took place between 1972 and 2005 there. Furthermore, the virus has been isolated continuously every year from 1984 to 1994 [35]. Our low transmission rate are difficult to reconcile with continuous ZIKV transmission, and suggest the involvement of other vectors or other mechanisms of maintenance and transmission. The involvment of other vectors is supported by the fact that lesser number of strains were isolated from the tested species compared to *Ae. furcifer* and *Ae. taylori* [35, 36]. Vector competence of these species should be investigated to better understand ZIKV epidemiology and transmission in Senegal. Our results suggest that the species tested here are probably less implicated in the regular ZIKV amplifications in Senegal. Other mechanisms of maintenance and transmission probably include the vertical and/or venereal transmission of the virus supported by its detection in several pools of male *Ae. furcifer* in nature [35, 36]. This phenomenon, already demonstrated in nature has never been proven experimentally and therefore needs further investigation.

## Conclusion

All the populations of *Ae. aegypti, Ae. unilineatus*, *Ae. vittatus*, and *Ae. luteocephalus* tested here were susceptible to oral infection of ZIKV but only a low proportion of *Ae. vittatus* and *Ae. luteocephalus* had the viral genome in their saliva and thus the potential to transmit the virus. Vector competence and vertical transmission studies involving other species more often associated with ZIKV should be undertaken to better understand the ecology and epidemiology of this arbovirus of growing medical interest.

## Additional file

Additional file 1: Table S1. Ct (threshold cycle) mean values for each mosquito species body (the whole body except legs and wings) and Zika virus strain at different days post-infection (dpi). (DOC 73 kb)

## Abbreviations

*Ae*.: *Aedes*
ATP: Adenosine 5’-triphosphate
dpi: days post-infection
FBS: Fetal bovine serum
IgM: Immunoglobuline M
NHPs: Non-human primates
PFU: Plaque forming unit
RH: Relative humidity
RNA: Ribonucleic Acid
RT-PCR: Reverse transcription-polymerase chain reaction
TCID_50_: 50 % tissue culture infectious dose
ZIKV: Zika virus
