# Supplementary material for: Potential of selected Senegalese Aedes spp. mosquitoes (Diptera: Culicidae) to transmit Zika virus
Source: BMC Infect Dis. 2015 Nov 2;15:492. doi: 10.1186/s12879-015-1231-2 (PMC4629289; doi:10.1186/s12879-015-1231-2)
Supplement: Additional file 1: Table S1. — Ct (threshold cycle) mean values for each mosquito species body (the whole body except legs and wings) and Zika virus strain at different days post-infection (dpi). (DOC 73 kb) [file 12879_2015_1231_MOESM1_ESM.doc]

Table S1: Ct (threshold cycle) mean values for each mosquito species body (the whole body except legs and wings) and Zika virus strain at different days post-infection (dpi).

|  |  |  | dpi | | |
| --- | --- | --- | --- | --- | --- |
| Species | ZIKV strains | Virus titer  (PFU/ml) | 5 | 10 | 15 |
| *Ae*. *aegypti* DKR | ArD 128000 | 4.6×10^6^ | 29.80 | 24.94 | 20.91 |
|  | ArD 132912 | 2×10^7^ | 38.88 | 27.10 | 25.22 |
|  | ArD 157995 | 5×10^6^ | 26.84 | 23.09 | 22.99 |
|  | ArD 165522 | 7.5×10^6^ | 21.54 | 23.70 | na |
|  | HD 78788 | 8.4×10^6^ | 30.47 | 20.76 | 21.93 |
|  | MR 766 | 10^7^ | 26.85 | 24.24 | 23.59 |
|  | All |  | 25.47 | 23.88 | 22.91 |
| *Ae*. *aegypti* KDG | ArD 128000 | 4.6×10^6^ | 24.77 | 21.77 | 20.24 |
|  | ArD 132912 | 2×10^7^ | 26.22 | 26.14 | 23.25 |
|  | ArD 157995 | 5×10^6^ | 23.22 | 21.15 | 20.53 |
|  | ArD 165522 | 7.5×10^6^ | 21.24 | 27.46 | 20.78 |
|  | HD 78788 | 8.4×10^6^ | 24.21 | 21.09 | 20.75 |
|  | MR 766 | 10^7^ | 22.79 | 22.58 | 22.46 |
|  | All |  | 23.70 | 22.69 | 21.68 |
| *Ae*. *luteocephalus* | ArD 128000 | 6.4×10^6^ | na | na | 19.76 |
|  | ArD 132912 | 5×10^6^ | na | na | 29.57 |
|  | ArD 157995 | 3×10^7^ | na | na | 19.52 |
|  | ArD 165522 | 9.1×10^6^ | na | na | 22.72 |
|  | HD 78788 | 8.7×10^6^ | na | na | 18.92 |
|  | MR 766 | 2×10^7^ | na | na | 20.98 |
|  | All |  | na | na | 20.58 |
| *Ae*. *unilineatus* | ArD 128000 | 6.4×10^6^ | 28.07 | 26.62 | 19.91 |
|  | ArD 132912 | 5×10^6^ | 30.27 | na | 19.30 |
|  | ArD 157995 | 3×10^7^ | na | 22.80 | 19.75 |
|  | ArD 165522 | 9.1×10^6^ | 30.61 | na | 22.56 |
|  | HD 78788 | 8.7×10^6^ | 27.36 | 26.71 | 26.06 |
|  | MR 766 | 2×10^7^ | 25.51 | 25.90 | 21.23 |
|  | All |  | 27.76 | 26.22 | 21.47 |
| *Ae*. *vittatus* | ArD 128000 | 4×10^7^ | 19.61 | 18.30 | 20.36 |
|  | ArD 132912 | 8.1×10^6^ | na | na | 5.77 |
|  | ArD 157995 | 10^7^ | 19.09 | 17.09 | 20.59 |
|  | ArD 165522 | 9.3×10^6^ | 22.49 | na | 22.79 |
|  | HD 78788 | 2.7×10^6^ | 15.05 | 18.04 | 18.42 |
|  | MR 766 | 3.3×10^7^ | 23.49 | 20.60 | 20.80 |
|  | All |  | 19.54 | 18.70 | 19.80 |
